# Evidence for tmTNF reverse signaling *in vivo*: Implications for an arginase-1-mediated therapeutic effect of TNF inhibitors during inflammation

**DOI:** 10.1016/j.isci.2021.102331

**Published:** 2021-03-21

**Authors:** Katy Diallo, Numa Simons, Souraya Sayegh, Michel Baron, Yannick Degboé, Jean-Frédéric Boyer, Andrey Kruglov, Sergei Nedospasov, Julien Novarino, Meryem Aloulou, Nicolas Fazilleau, Arnaud Constantin, Alain Cantagrel, Jean-Luc Davignon, Benjamin Rauwel

**Affiliations:** 1 INFINITy, Toulouse Institute for Infectious and Inflammatory Diseases, INSERM U1291, CNRS U5051, University Toulouse III, Toulouse, France; 2 Centre de Rhumatologie, CHU de Toulouse, Toulouse, France; 3 Faculté de Médecine, Université Paul Sabatier Toulouse III, Toulouse, France; 4 German Rheumatism Research Center (DRFZ), a Leibniz Institute Berlin 10117, Germany; 5 Engelhardt Institute of Molecular Biology, Russian Academy of Sciences, Moscow 119991, Russia

**Keywords:** Immunology, Cell Biology

## Abstract

In order to ascertain the significance of transmembrane tumor necrosis factor (tmTNF) reverse signaling *in vivo*, we generated a triple transgenic mouse model (3TG, TNFR1−/−, TNFR2−/−, and tmTNFKI/KI) in which all canonical tumor necrosis factor (TNF) signaling was abolished. In bone-marrow-derived macrophages harvested from these mice, various anti-TNF biologics induced the expression of genes characteristic of alternative macrophages and also inhibited the expression of pro-inflammatory cytokines mainly through the upregulation of arginase-1. Injections of TNF inhibitors during arthritis increased pro-resolutive markers in bone marrow precursors and joint cells leading to a decrease in arthritis score. These results demonstrate that the binding of anti-TNF biologics to tmTNF results in decreased arthritis severity. Collectively, our data provide evidence for the significance of tmTNF reverse signaling in the modulation of arthritis. They suggest a complementary interpretation of anti-TNF biologics effects in the treatment of inflammatory diseases and pave the way to studies focused on new arginase-1-dependent therapeutic targets.

## Highlights

• *In vivo* demonstration of tmTNF reverses signaling existence
• tmTNF reverse signaling induces anti-oxidative stress response
• tmTNF reverse signaling induces an arginase-1-mediated anti-inflammatory response
• Reverse signaling is a complementary mechanism to TNF neutralization by anti-TNF

## Graphical abstract

**Figure:**
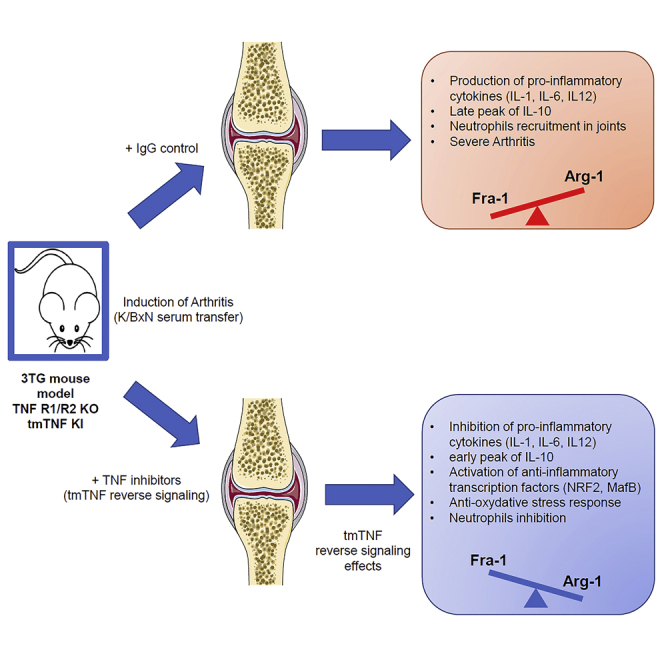

## Introduction

Tumor necrosis factor (TNF) is a homotrimeric pro-inflammatory and immunomodulatory cytokine. Like most members of the TNF superfamily, it exists either in a transmembrane form (transmembrane tumor necrosis factor [tmTNF]) or in a soluble form (soluble tumor necrosis factor [sTNF]) after cleavage of its precursor—(tmTNF)—by the protease TACE (TNF alpha converting enzyme, ADAM17). Both forms are bioactive (Moss et al., 1997; Zheng et al., 2004). Once secreted, sTNF acts in an autocrine or paracrine manner by binding to either one of its two receptors, TNFR1 and TNFR2 (Szondy and Pallai, 2017), which both also exist in transmembrane and soluble forms. TNFR1 is ubiquitously expressed on most cells and its stimulation leads to the activation of pro-inflammatory or apoptotic/necroptotic pathways depending on the context. TNFR2 is an inducible receptor whose expression is mostly restricted to immune, endothelial, and neuronal cells with a higher affinity for tmTNF (Grell et al., 1995). In addition to its pro-inflammatory role, TNFR2 could also act as an anti-inflammatory mediator and as such plays a role in cell signaling and promotes cell survival (Wajant et al., 2003).

Rheumatoid arthritis (RA) is a chronic inflammatory autoimmune disease that affects 0.5% of the adult population. It is characterized by the infiltration of synovial compartment of joints by immune cells (Smolen et al., 2016). Macrophages play a central role in the induction and maintenance of chronic inflammation during various stages of the disease (Siouti and Andreakos, 2019; Tak, 2000). One of the main mechanisms involved in this process is the secretion of pro-inflammatory cytokines such as interleukin (IL)-1β, IL-6, and TNF. The latter plays a leading role in the activation of immune responses through the production of inflammatory mediators but also by stimulating osteoclastogenesis (Butler et al., 1995; Haworth et al., 1991; Kitaura, 2005).

The development of TNF inhibitors has permitted to control the activity of RA in a substantial proportion of patients (Feldmann et al., 2010). There are currently five different molecules available, two human anti-TNF monoclonal antibodies (adalimumab, golimumab), a pegylated antigen-binding fraction of humanized antibodies (certolizumab pegol), a murine chimeric antibody (infliximab), and a soluble form of TNFR2 coupled to human Fc domain (etanercept [ETA]) (Taylor and Feldmann, 2009).

Unfortunately, these treatments are not always effective, with an estimated 40% of unsatisfactory responses to one of these biologics. TNF antagonists are thought to control inflammation by preventing the binding of sTNF to its receptors and are all equally effective in lowering TNF concentrations (Kaymakcalan et al., 2009; Mitoma et al., 2018; Nesbitt et al., 2007). However, they are also all able to bind tmTNF (Horiuchi et al., 2010). It has been demonstrated that tmTNF can act *in vitro* as a signaling receptor through a mechanism known as “reverse signaling” (Eissner et al., 2000) which may play a role in cellular communications (Eissner et al., 2004).

Specifically, TNFR2 was shown to interact with tmTNF and induce intra-cellular responses through reverse signaling that contributes to an increased lipopolysaccharides (LPS) resistance, via the activation of the mitogen-activated protein kinase/extracellular signal-regulated kinase pathway (Boyer et al., 2007; Eissner et al., 2000; Kirchner et al., 2004; Mitoma et al., 2005; Pallai et al., 2016). Furthermore, the involvement of nuclear factor erythroid-2-related factor 2 (Nrf2) activation during reverse signaling was recently discovered in our laboratory (Boyer et al., 2016). More recently, internalization of anti-TNF antibody/tmTNF complexes by dendritic cells has been demonstrated. The authors have explored anti-drug-antibody genesis but other consequences of such internalization, including reverse signalization, can be also envisioned (Deora et al., 2017).

Several studies implicated a role for reverse signaling in various biological processes including *in vitro* neuronal growth (Kisiswa et al., 2013), macrophage inflammation (Meusch et al., 2009), and apoptosis (Meusch et al., 2013). However, the significance of tmTNF reverse signaling *in vivo* has yet to be demonstrated.

The K/BxN model induces peripheral arthritis phenotypically similar to RA in mice (Korganow et al., 1999; Kouskoff et al., 1996). Mice injected with K/BxN serum develop peripheral arthritis, which relies solely on innate immunity (macrophages, polynuclear neutrophils, complement) and more specifically on neutrophils (Ji et al., 2002a; Wipke and Allen, 2001), is partially dependent on TNF (Ji et al., 2002b), and resolves within 14–21 days.

No model was available to define TNF reverse signaling *in vivo* and to study the impact of tmTNF reverse signaling on macrophage polarization and inflammation.

To this end, we generated a triple transgenic mouse model (3TG) lacking TNFR1 and TNFR2 expression (TNFR1/R2 KO) and expressing TNF at a physiological level exclusively in its transmembrane form (tmTNF KI) due to knock-in mutations (Ruuls et al., 2001). These 3TG mice were developed as an experimental model to simplify the study of tmTNF reverse signaling *in vitro* but also *in vivo*. The comparison with wild type (WT) animals is not the aim of this study and will be discussed later. In primary bone-marrow-derived macrophages (BMDMs) from these mice, we evaluated the effects of anti-TNF stimulation on macrophage polarization *in vitro*. We observed that tmTNF reverse signaling induced a decrease of the pro-inflammatory transcription factor Fra-1 and consequently an upregulation in pro-resolutive transcription factors and effectors such as arginase-1 (Arg-1). Furthermore, we observed that tmTNF reverse signaling induced an early peak of IL-10 and inhibited pro-inflammatory cytokines, such as IL-6 and IL-12. Finally, using the K/BxN serum injection arthritis model in WT and 3TG mice, we showed that anti-TNF injections induced an increase in pro-resolutive transcription factor and enzyme (Arg-1) in bone marrow precursors and inhibited arthritis. Furthermore, pro-inflammatory IL-1β was inhibited, and neutrophils were less numerous in the joints of treated mice, thus demonstrating for the first time the significance of reverse signaling *in vivo* and implicating a novel interpretation of the effects of anti-TNF therapy in the treatment of inflammatory diseases, such as RA.

## Results

### Internalization of soluble TNF receptor 2 (ETA) through its interaction with tmTNF suggests reverse signaling in macrophages

We first validated our mouse model specifically developed to study reverse signaling. By flow cytometric analysis of immune cell populations in the spleen and inguinal lymph nodes, we did not observe significant differences in these cell populations between WT and 3TG mice (Figures S1A–S1C). As expected, secretion of TNF was null in 3TG mice (Figure S1D). The expression of tmTNF before and after stimulation with LPS + IFN-γ was similar in BMDMs from WT and 3TG mice, suggesting an equivalent capacity to interact with its ligands (Figure S1E).

**Figure 1:**
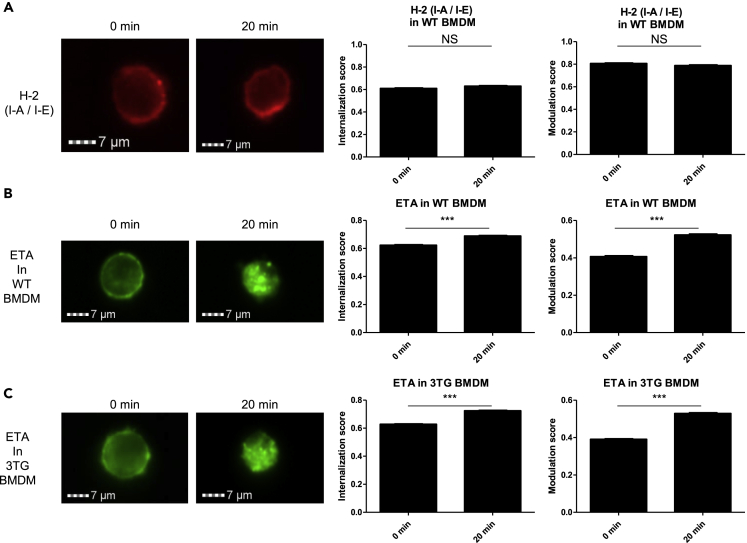
Internalization of soluble TNF receptor 2 (ETA) through its interaction with tmTNF suggests reverse signaling in macrophages Non-polarized BMDMs were stimulated with LPS (50ng/mL) 30 min prior to staining in presence of Fc blocker with H-2-PE-Cy5 in WT (A) or ETA-dyelight488 in WT (B) and 3TG (C) during 20 min at 4° (0 min) or 37°C (20min). Internalization and modulation scores were analyzed by imaging cytometry (ISX). Data are presented as mean ± SEM of internalization or modulation scores (∗∗∗p < 0.001, n > 500 events, Student's t test). Images are representative of 3 independent experiments with more than 500 events analyzed each time.

We then investigated the interaction of soluble TNF receptor 2 (ETA) with tmTNF in BMDMs from 3TG mice. To this end, we performed imaging cytometry on BMDMs after 30 min of stimulation with LPS (50 ng/mL) as a mean to increase tmTNF cell surface expression. Cells were then incubated with ETA conjugated to DyeLight488 (ETA-488), and an internalization assay was performed. As a control, we used anti-H-2 (I-A/I-E) phycoerythrin-conjugated antibody which did not induce any internalization (Figure 1A). Higher internalization and modulation score at 20 min indicate that ETA-488 was internalized in WT (Figure 1B) as well as in 3TG BMDMs (Figure 1C). This internalization was observed in the presence of an Fc-blocker suggesting that it was mediated by interaction with tmTNF and not through Fc receptor. Furthermore, higher internalization was observed in 3TG in comparison with WT (0.68 vs 0.73, p < 0.001), suggesting that in the absence of sTNF, enhanced interaction of ETA-488 with tmTNF was detected. Similar results were obtained with rat anti-murine-TNF antibody (MP6-XT22) in WT and 3TG cells (Figures S2A and S2B). These results demonstrated an internalization of soluble TNF receptor 2 (ETA) and suggested as a consequence of tmTNF/anti-TNF interaction, an ETA-mediated reverse signaling in macrophages as observed in our laboratory with certolizumab pegol (Boyer et al., 2016).

### Impact of tmTNF reverse signaling on macrophage polarization *in vitro*

TNF inhibitors are known to modulate macrophage polarization (Degboé et al., 2019). We thus assessed the effects of tmTNF reverse signaling on macrophage phenotypes. BMDMs were harvested from WT and 3TG mice and cultured with or without a control IgG (CTRL), an anti-murine-TNF antibody (MP6-XT22) or a soluble TNF-R2 (ETA) during their differentiation. After 7 days, BMDMs were polarized into a pro-inflammatory phenotype by adding LPS/IFN-γ for 24 hr. Using flow cytometry analysis, we observed that anti-TNF antibody but not ETA inhibited the expression of a panel of pro-inflammatory markers during BMDM differentiation and M1 polarization. Indeed, MP6-XT22 treatment led to a decrease of Ly6C and CD40 expression in WT cells. However, this inhibition was not observed in 3TG cells, suggesting that this effect was not mediated by reverse signaling (Figure S3A–S3C). No difference was observed in CD80, GPR18, CD38, and FPR2 expression levels in anti-TNF-treated WT and 3TG BMDMs (Figures S3D–S3G). When we focused on the pro-resolutive markers CD163 and CD206, no modulation was detected between cells obtained from WT or 3TG mice treated with anti-TNF and those that were not treated (Figure S3H and S3I). No effect was observed in presence of rat control IgG (Figure S4)

**Figure 2:**
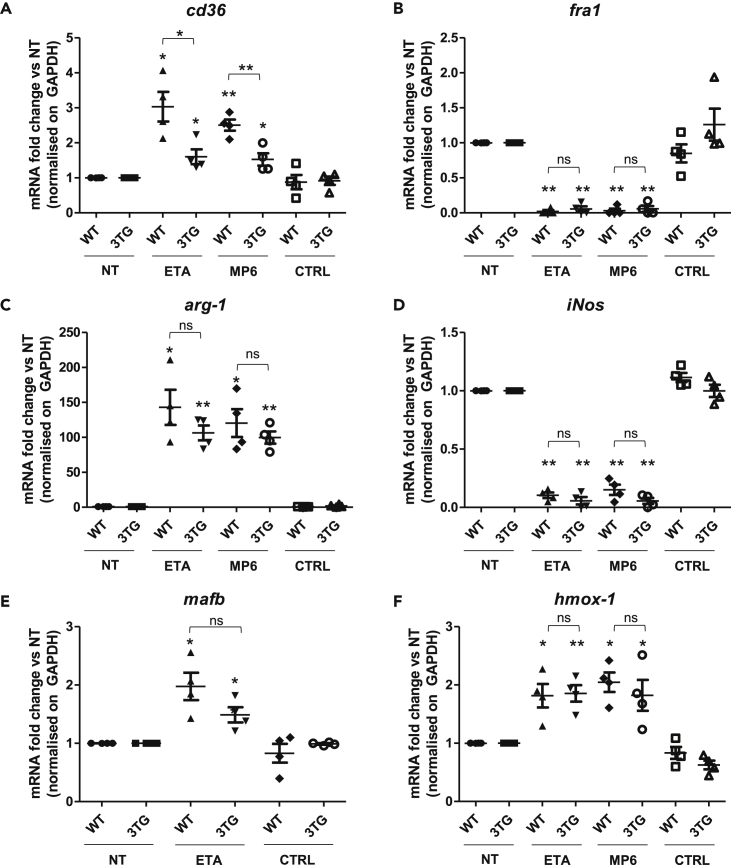
tmTNF reverse signaling phenotypic impact on macrophage polarization *in vitro* WT or 3TG BMDMs were obtained after 7 days of differentiation with recombinant M-CSF (50 ng/mL) in the presence or absence (NT) of 10 μg/mL of TNF soluble receptor (ETA), anti-TNF antibody (MP6-XT22), or IgG control (CTRL). BMDMs were then polarized into pro-inflammatory M1 macrophage during 24 hr with LPS (100 ng/mL) and IFN-γ (25 ng/mL) in the presence or not (NT) of fresh ETA, MP6-XT22, or CTRL. RNA were extracted and *cd36* (A), fra*-1* (B), *arg1* (C), *iNos* (D), *mafb* (E), and *hmox-1* (F) mRNA expression was analyzed by RT-qPCR in 3TG and WT BMDMs. Data are presented as mean ± SEM of mRNA fold change vs NT normalized on *gapdh*. (n = 4, ns p > 0.05, ∗p < 0.05, ∗∗p < 0.01, Mann-Whitney U test performed).

Then, we analyzed the expression of the scavenger receptor CD36 which was previously shown to be upregulated by anti-TNF treatment in human monocytes (Boyer et al., 2007). In our BMDM model, we observed a significant increase of the CD36 surface (Figure S5A) and mRNA expression (Figure 2A) in WT as well as in 3TG cells, suggesting that it was mediated by reverse signaling. These results demonstrated that only anti-TNF antibodies inhibit the expression of pro-inflammatory markers Ly6C and CD40 but both antibodies and ETA promote pro-resolutive marker CD36. However, tmTNF reverse signaling was only involved in the modulation of CD36 expression.

We next sought to evaluate the impact of tmTNF reverse signaling on the expression of functional effectors of pro-inflammatory and pro-resolutive macrophages. The pro-inflammatory effects of Fra-1 are controlled through the balance of Fra-1/Arg-1 (Hannemann et al., 2019). *In vitro*, we observed a strong decrease in Fra-1 expression in WT and 3TG M1 macrophages in the presence of anti-TNF but not in the IgG control-treated cells (Figure 2B). Consistent with Fra-1 decrease, a strong increase of Arg-1 mRNA and protein was confirmed in anti-TNF treated cells (Figures 2C and S5B), thus reversing the balance of Fra-1/Arg-1 (Figure S5C). Furthermore, expression of the pro-inflammatory enzyme Nos2, also involved in the L-arginine pathway, was inhibited by anti-TNF in comparison with the IgG control (Figure 2D). These observations lead to the analysis of MafB, a pro-regulator of Arg-1 and anti-inflammatory transcription factor in macrophages (Kim, 2017). We demonstrated that MafB mRNA was increased after stimulation by TNF soluble receptor in WT and 3TG BMDMs (Figure 2E), and this correlated with an increase of MafB protein in the anti-TNF stimulated BMDMs (Figure S5D), consistent with the upregulation of Arg-1 and anti-inflammatory macrophage polarization. We could also detect an upregulation of the transcription factor C-myc (Figure S5E), although this upregulation was not significant in WT BMDMs. No significant differences were observed in regard to the pro-resolutive transcription factors Mrc-1 and Egr-2 (Figures S5F and S5G).

We also recorded an upregulation of the anti-oxidative stress response genes, *hmox-1* (Figure 2F) and *gclc* (Figure S5H). These effectors are under the control of the anti-inflammatory NRF2 which we had previously linked to reverse signaling in human monocytes (Boyer et al., 2016). No effect was observed in presence of rat IgG control (Figure S5I). All these modulations in favor of pro-resolutive effectors were detected with similar intensity in WT and 3TG BMDMs, except for *cd36* where the upregulation is higher in WT. These results indicate that tmTNF reverse signaling may impact macrophage polarization in favor of pro-resolutive functions.

### tmTNF reverse signaling induces an early peak of IL-10 secretion and inhibits pro-inflammatory cytokines *in vitro*

**Figure 3:**
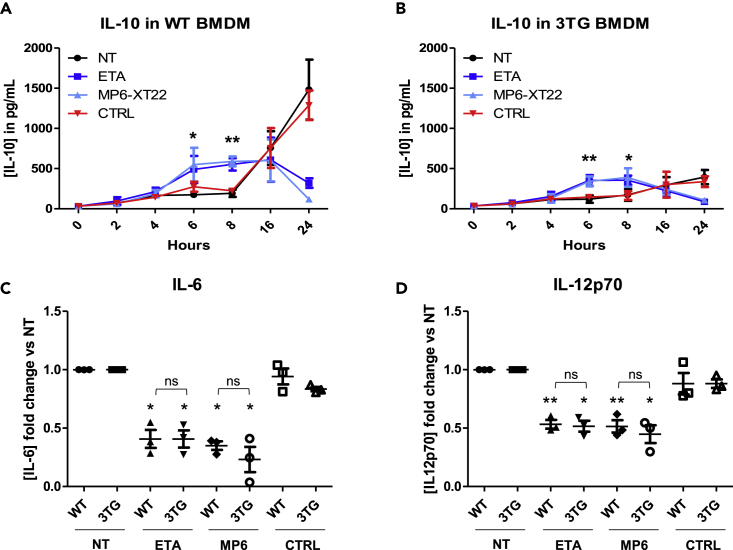
tmTNF reverse signaling induces an early peak of IL-10 secretion and inhibits pro-inflammatory cytokines *in vitro* WT or 3TG BMDMs were obtained during 7 days of differentiation with recombinant M-CSF (50 ng/mL) in presence or absence (NT) of TNF soluble receptor (ETA), anti-TNF antibody (MP6-XT22), or IgG control (CTRL) prior to being polarized into pro-inflammatory M1 macrophage during 24 hr with LPS (100 ng/mL) and IFN-γ (25 ng/mL) in presence or not (NT) with fresh ETA, MP6-XT22, or CTRL. Twenty-four hour secretion kinetic of IL-10 in M1 polarized in WT(A) and 3TG (B) BMDM was analyzed by ELISA. IL-10 concentration at 6 hr and 10 hr after stimulation by LPS-IFN-γ is represented in histograms. After 24 hr of M1 polarization, IL-6 (C) and IL12p70 (D) concentrations were analyzed by cytometric bead array. Data represent mean ± SEM of cytokine concentration fold change versus NT (n = 3, ∗p < 0.05, ∗∗p < 0.01, Mann-Whitney U test). ELISA, enzyme-linked immunosorbent assay.

To investigate the consequences of tmTNF reverse signaling-mediated upregulation of pro-resolutive effectors, cytokine production was measured during LPS/IFNγ-mediated M1 polarization in the presence of TNF inhibitors. When control Ab were used, production of IL-10 in cells from WT mice increased over a 24 hr time period, which reflected the negative feedback of TNF-induced inflammation (Sabat et al., 2010) (Figure 3A). In contrast, in cells from 3TG mice, production of IL-10 was modest, due to the absence of TNF-induced negative feedback (Figure 3B). Treatment with anti-TNF and ETA decreased the production of IL-10 in WT cells at the 24^th^ hour and in 3TG cells at the 16^th^ hour of culture. Confirming our observations in human macrophages (Degboé et al., 2019), TNF inhibitors induced an early peak of IL-10 secretion in WT (Figure 3A) and 3TG (Figure 3B) after 6 hr of stimulation. As no TNF receptors are present in 3TG mice, this early production of IL-10 is likely to result from tmTNF reverse signaling. Consistent with this early peak of IL-10 secretion, we observed a strong decrease in IL-6 (Figure 3C) and IL-12p70 (Figure 3D) in anti-TNF-treated cells compared to controls. IL-1β and TGF-β were not detectable in cell supernatants. These results indicate that tmTNF reverse signaling induces a rapid upregulation of IL-10 secretion coupled to an inhibition of IL-6 and IL-12 production.

### tmTNF reverse signaling anti-inflammatory effect is mainly mediated by arginase-1

**Figure 4:**
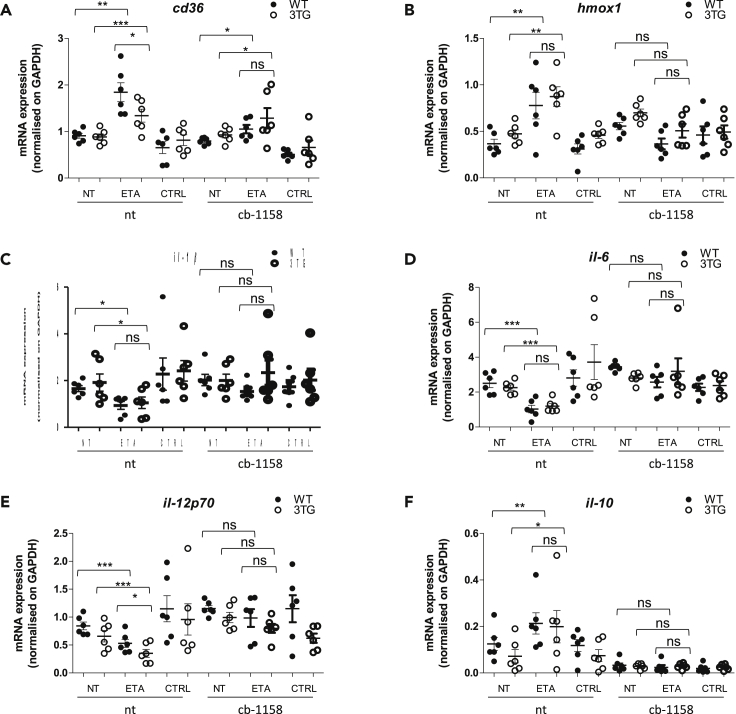
tmTNF reverse signaling anti-inflammatory effect is mainly mediated by ARG-1 WT or 3TG BMDMs were obtained during 7 days of differentiation with recombinant M-CSF (50 ng/mL) in presence or absence (NT) of TNF soluble receptor (ETA), anti-TNF antibody (MP6-XT22) or IgG control (CTRL), and arginase inhibitor (CB-1158; 10 μM) prior to being polarized into pro-inflammatory M1 macrophage during 24 hr with LPS (100 ng/mL) and IFN-γ (25 ng/mL) in presence or not (NT) with fresh ETA, MP6-XT22 or CTRL, and CB-1158. RNAs were extracted and *cd36* (A), *hmox-1* (B), *il-1β* (C), *il-6* (D), *il-12p70* (E), and *il-10* (F) mRNA expression was analyzed by RT-qPCR in 3TG and WT BMDMs. Data are presented as mean ± SEM of mRNA fold change vs NT normalized on *gapdh*. (n = 6, ∗p < 0.05, ∗∗p < 0.01, unpaired t test performed).

To analyze the impact of tmTNF reverse signaling-mediated arginase-1 upregulation on pro-inflammatory cytokine inhibition, we used CB-1158, a specific arginase inhibitor which has been shown to block myeloid cell-mediated immune suppression (Steggerda et al., 2017). BMDMs were treated with CB-1158 during differentiation and polarization with or without ETA or control IgG. We observed that *cd36* upregulation was not significantly modulated by the inhibition of Arg-1 (Figure 4A) in contrary to *hmox-1* upregulation observed with ETA treatment which was inhibited in the presence of Arg-1 inhibitor (Figure 4B). When we focused on pro-inflammatory cytokines, we revealed that ETA-mediated downregulation of *il-1β*, *il-6*, and *il-12* was less efficient when Arg-1 was inhibited (Figures 4C–4E). Finally, we observed that *il-10* upregulation by anti-TNF was also inhibited in the presence of CB-1158. All these modulations were observed with similar intensity in WT and 3TG BMDMs. These results suggested that tmTNF reverse anti-inflammatory effect is mainly mediated by the upregulation of Arg-1.

### tmTNF reverse signaling role in the therapeutic response to TNF inhibitors during arthritis

**Figure 5:**
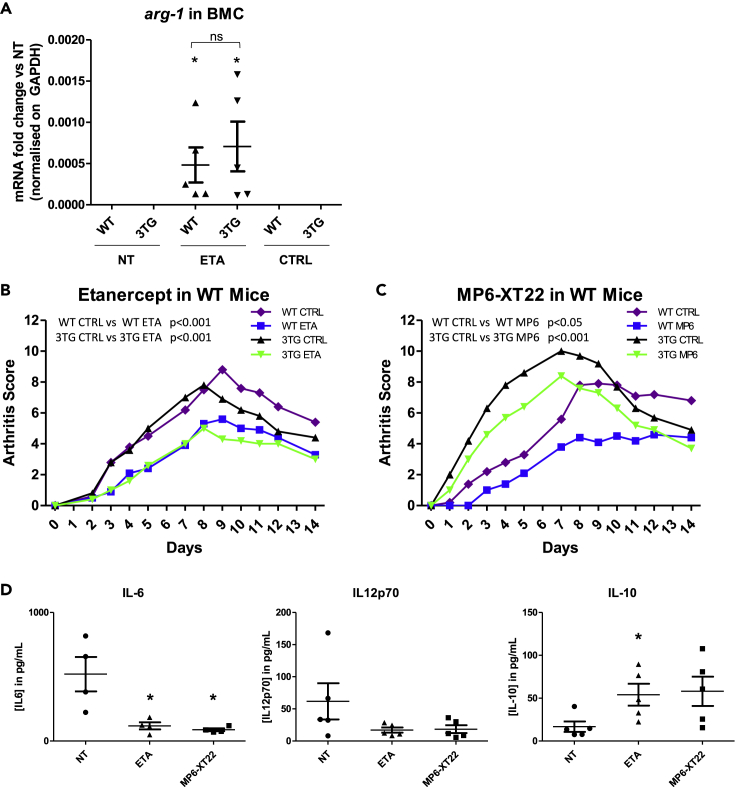
tmTNF reverse signaling role in the therapeutic response to TNF inhibitors during arthritis Arthritis was induced by K/BxN serum transfer in 3TG or WT mice treated or not with anti-TNF (ETA or MP6-XT22) or control IgG1 (CTRL). (A) At day 0, the bone marrow from 4 WT or 3TG mice of each group (NT, ETA, and CTRL) was collected and mRNA from precursor cells was extracted. Expression of *Arg-1* was analyzed by RT-qPCR. Data represent mean ± SEM of mRNA expression normalized on *gapdh* expression (n = 4). Statistics analysis was performed with Mann-Whitney U test. ND = non detectable. (B and C) Clinical effect of (B) TNF soluble receptor 2 (etanercept, ETA, 10 mg/kg) or (C) anti-mouse TNF rat antibody (MP6-XT22, 10 mg/kg) on the development of arthritis (arthritic score) in the WT or 3TG K/BxN serum-transferred mice (n = 5 per group). Control (CTRL): untreated K/BxN serum-transferred mice. Results are presented as mean arthritic score during 14 days after K/BxN injection. Data represent mean ± SEM. p value for arthritis score was calculated by repeated measurements of two-way ANOVA tests. (D) Concentrations of IL-6, IL-12p70, and IL-10 in blood samples of 3TG arthritic mice were quantified by cytometric bead array 7 days after K/BxN first injection. Data represent mean ± SEM of cytokine concentrations (n = 5, except for IL-6, n = 4. ∗p < 0.05 as calculated with Mann-Whitney U test).

**Figure 6:**
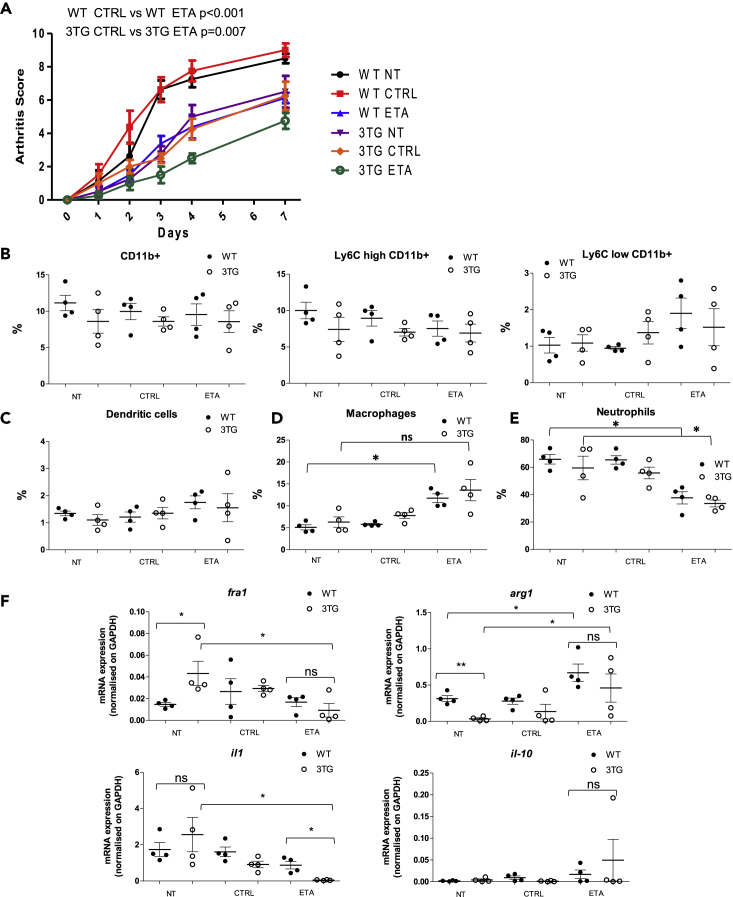
tmTNF reverse signaling impact on immune cells and inflammation in arthritic joints Eight-week-old 3TG or WT mice were injected at days 0 and 2 intraperitoneally with 200 μL of 60-week-old K/BxN mice serum to induce arthritis. Mice were injected with 10 mg/kg of anti-TNF (ETA) or control IgG1 (CTRL) 5 and 3 days prior to inducing arthritis with the first injection of KBxN serum and at days 0, 2, 4, and 7. Mice were sacrificed at day 7, and joints were dissected. (A) Clinical effect of ETA or CTRL on the development of arthritis (arthritic score) in the WT (left panel) or 3TG (right panel) K/BxN serum-transferred mice (n = 4 per group). Control (CTRL): untreated K/BxN serum-transferred mice. Results are presented as mean arthritic score during 7 days after K/BxN injection. Data represent mean ± SEM. p value for arthritis score was calculated by repeated measurements of two-way ANOVA tests. (B–E) Flow cytometry analysis of percentage of monocytes (B), dendritic cells (C), macrophages (D), and neutrophils (E) in joints. (F) RT-qPCR analysis of *Fra-1, Arg-1, IL-1β*, and *IL-10* mRNA expression. Data represent mean ± SEM of percentage of leaving cells or mRNA expression normalized on GAPDH (n = 4, ns p > 0.05, ∗p < 0.05 as calculated with Mann-Whitney U test).

Finally, we studied the role of tmTNF reverse signaling in the therapeutic response to anti-TNF therapy during arthritis. Eight-week-old 3TG or WT mice were pre-injected with either anti-TNF or control IgG 5 and 3 days prior to inducing K/BxN serum transfer arthritis as described in our experimental protocol (Figure S6A). At day 0, bone marrow precursor cells from femurs and tibias were collected, and the expression of pro-inflammatory and pro-resolutive markers was analyzed by reverse transcription quantitative polymerase chain reaction (RT-qPCR). Injection of anti-TNF or ETA upregulated the expression of pro-resolutive effectors Arg-1, Mrc-1, and Egr-2 in precursors cells *in vivo* in WT as well as in 3TG cells (Figures 5A, 5B, S6B, and S6C). Fra-1 mRNA was not detectable in those precursor cells, and no significant differences for c-myc and CD38 expression were observed (Figures S6B and S6C). These results argue in favor of tmTNF reverse signaling *in vivo* and its impact on pro-resolutive effector expression. At day 0 and 2, mice were intraperitoneally injected with K/BxN serum to induce arthritis. Animals received control IgG or anti-TNF injection at day 0, 2, 4, 7, 9, and 11. Evolution of arthritis was evaluated over 14 days. We observed that ETA injection in WT mice decreased the arthritis score over the course of the experiment (p < 0.001, two-way analysis of variance [ANOVA] test) (Figure 5C). Although the severity of arthritis was lower, inhibition of arthritis was also observed in 3TG mice (Figure 5D) (p < 0.001, two-way ANOVA test), demonstrating that this effect was indeed due to tmTNF-mediated reverse signaling, which was confirmed using an anti-TNF antibody (MP6-XT22). This antibody significantly lowered the clinical score in WT as well as in 3TG mice (Figure 5D). There was no significant effect of control IgG injection on the arthritis cumulative score (Figures S7A and S7B), further proving that the effects of anti-TNF injection were due to tmTNF reverse signaling and were not mediated by Fc-receptors. Cytokine concentrations were measured in blood samples at the peak of arthritis (day 7). As expected, in contrary of WT mice, no sTNF was detected in 3TG mice (Figure S7C). Nevertheless, we recorded a significant decrease in IL-6 and a trend toward a decrease of IL-12 in 3TG mice (Figure 5E). IL-1β was not detectable in sera. IL-10 increased after anti-TNF treatment but was statistically significant only with ETA (Figure 5E). This lack of significance was probably due to a lower impact of anti-TNF in peripheral blood than in arthritis. To focus on the effect of reverse signaling in joints, mice treated or not with ETA or IgG1 control were sacrificed at the peak of arthritis (Figure 6A). By flow cytometry analysis of joint cell populations, we could observe that anti-TNF treatment did not have any significant impact on total monocytes, even if a trend toward an upregulation of pro-resolutive Ly6c^low^ Cd11b^+^ monocytes was recorded in WT ETA-treated mice as observed in BMDMs *in vitro* (Figure 6B). No effect on dendritic cells (Figures 6C) and B lymphocytes (Figure S7D) was noted. We observed an increase in macrophages only in treated WT mice (Figure 6D), suggesting that this effect is principally due to sTNF neutralization. Nevertheless, the principal effectors in K/BxN serum transfer arthritis model, neutrophils (Wipke and Allen, 2001), were decreased in both WT and 3TG ETA-treated mice joints (Figure 6E). These results demonstrate the anti-inflammatory effect of reverse signaling, strengthened by the observed upregulation of Arg-1 and IL-10 mRNA coupled with the downregulation of pro-inflammatory Fra-1 and IL-1β expression in WT as in 3TG joints (Figure 6F), although no significant effect was observed on IL-6 and IL-12 mRNA expression (Figures S7E and S7F). Thus, our experiments strongly support an anti-inflammatory role for reverse signaling *in vivo* and its implications in the therapeutic response to anti-TNF during arthritis.

## Discussion

In this work, we present evidence for *in vivo* anti-TNF-induced tmTNF reverse signaling and demonstrate for the first time its impact on macrophage polarization and its functional role in the clinical response to TNF blocking therapy during arthritis *in vivo*.

Contrary to what was previously published (Nguyen et al., 2018), we observed that ETA binds to tmTNF and can be internalized by macrophages. This observation suggests that ETA as well as anti-TNF antibody is capable of inducing a reverse signaling through tmTNF. We could hypothesize that this internalization will induce an increase of reactive oxygen species which be responsible of NRF2 activation as previously described (Boyer et al., 2016) or will induce an outside-to-inside signaling similar or different of the previously described for anti-TNF antibodies (Mitoma et al., 2005). Studying the complete mechanism of reverse signaling is a story in itself and will be the subject of future work.

*In vitro*, we saw that TNF blockade during WT macrophage differentiation, by antibodies but not ETA, inhibited the expression of pro-inflammatory surface markers, such as CD40 and Ly6c. Such inhibition can be attributed to the neutralization of sTNF as it was not observed in our 3TG model. This difference between antibody and ETA may be due to a different interaction with sTNF and tmTNF (Kohno et al., 2007; Scallon et al., 2002). As opposed to human macrophage polarization by TNF inhibitors (Degboé et al., 2019), we did not see any modulation of CD163 and CD206 in murine macrophages. Those effects might be specific to human macrophages or might be due to the source of cells used (blood vs bone marrow). However, in 3TG as well as in WT BMDM, we observed an increase in the expression of scavenger receptor CD36- and NRF2-dependent anti-oxidative stress response genes, thus proving and confirming that CD36 modulation and NRF2 activation in response to anti-TNF is due to tmTNF-mediated reverse signaling (Boyer et al., 2016).

From a functional point of view, by studying the transcription factors in addition to the key enzymes of the induction/maintenance or the resolution of inflammation, we concluded that tmTNF reverse signaling induced a pro-resolutive switch to the detriment of certain pro-inflammatory actors. Indeed, we observed in our 3TG model as in WT BMDMs that the reverse signaling inhibited the expression of the transcription factor Fra-1 and increased the pro-resolutive enzyme Arg-1 expression. Recently, it has been shown that this Fra-1/Arg-1 balance is related to the severity of RA (Hannemann et al., 2019). It has already been described that soluble TNF neutralization could increase Arg-1 and pro-resolutive macrophage differentiation (Kroner et al., 2014; Kratochvill et al., 2015; Atretkhany et al., 2016; Schleicher et al., 2016). Nevertheless, with the help of our 3TG model, we demonstrated for the first time that tmTNF reverse signaling is also able to do it. This suggest that there is a complementary effect of reverse signaling and soluble cytokine neutralization that could explain the significant differences between WT and 3TG animals. Indeed, TNF blocker effects were always more efficient in WT animals, due to the combination of sTNF neutralization and tmTNF reverse signaling. More interestingly, we noticed that this upregulation of Arg-1 is correlated with the increase of mRNA and nuclear protein expression of MafB, a positive regulator of Arg-1 which is able to bind to its promoter and regulate a pro-resolutive polarization of macrophages (Kim, 2017). We also evidenced that the inhibition of pro-inflammatory cytokines and anti-oxidative stress response of tmTNF reverse signaling is principally mediated by this upregulation of Arg-1. In addition, we observed an increase in pro-resolving transcription factors such as c-myc, MRC-1, or Egr2 as well as an inhibition of the M1 Nos2 enzyme.

As for cytokine secretion, an early peak of IL-10 was noticed, similar to human macrophages cultured in the presence of TNF inhibitors (Degboé et al., 2019). This IL-10 early peak is then followed by an inhibition of pro-inflammatory cytokine production. As previously described in Degboé’s work, anti-inflammatory effects and pro-resolutive macrophage differentiation of TNF blockers are more efficient when they are added during all the differentiation and polarization time (data not shown) in contrary of polarization alone. This suggests that the TNF blocker effects in patients are not only immediate but also over time. Thus, our data provide conclusive evidence that TNF targeting is able, through tmTNF reverse signaling, to inhibit the secretion of pro-inflammatory cytokines.

Furthermore, in line with the anti-inflammatory effect observed *in vitro*, we demonstrated that tmTNF reverse signaling inhibits arthritis *in vivo*. The low TNF dependence of K/BxN serum transfer models has allowed us to induce arthritis in our 3TG model. Injection of anti-TNF during K/BxN serum-induced arthritis decreased clinical score in WT as in 3TG mice, suggesting a crucial role of tmTNF reverse signaling in the therapeutic response to anti-TNF biologics. We had to use 2 pre-injections of ETA or MP6-XT22 as it has been described that ETA does not have an impact in WT mice in K/BxN serum transfer models without pre-injection (Victoratos and Kollias, 2009), certainly due to the fast kinetic of this model. Although we used a prophylactic treatment model, the interest of our study is focused on the demonstration of the existence of tmTNF reverse signaling *in vivo* and its crucial role in the anti-inflammatory effect of anti-TNF agents. Since patients have constant circulating anti-TNF levels during treatment, it is also conceivable that this prophylactic effect may be found in cells which are differentiating during the disease. To better understand the consequences of tmTNF reverse signaling in arthritis, we focused on joint cell populations and discovered that anti-TNF treatment decreased localized inflammation. Indeed, in anti-TNF treated WT and 3TG joints, neutrophil population was inhibited and this was accompanied by a decrease of pro-inflammatory IL-1β expression. This inhibition of neutrophils could be due to direct tmTNF reverse signaling as they express TNF and logically tmTNF or due to the neutralization of TNF that could not induce pro-survival signals in these cells (Cowburn et al., 2004; Wright et al., 2011). Furthermore, increases of pro-resolutive effectors (IL-10 and ARG-1) were also observed, strengthening the anti-inflammatory effect of tmTNF reverse signaling.

The demonstration that anti-TNF agents can ameliorate arthritis by their action on tmTNF leads to a new interpretation of the effects of anti-TNF therapy in RA and other inflammatory diseases. We discovered novel mechanisms of action of these drugs, including the activation of MafB and NRF2 transcription factors and the increase of the anti-inflammatory regulator IL-10 and Arg-1. We can hypothesize that the intra-individual variability in anti-TNF clinical responses depends in part on the proteins recruited during the formation and internalization of the tmTNF/anti-TNF complexes (Deora et al., 2017; Ogura et al., 2016) and may be linked to specific epitopes recognized by the anti-TNF biologics used. Setting apart the roles of tmTNF reverse signaling on the one hand and the inhibition of sTNF on the other hand will help to better understand the mechanisms of action of TNF inhibitors.

Finally, this 3TG model permits us to highlight the effect of tmTNF reverse signaling on inflammation and oxidative stress response in the absence of TNFR1/R2 expression and a mutated tmTNF (deletion/mutation combination) with normal cell-surface expression and function which avoid an interaction with other receptors (Decoster et al., 1995). Nevertheless, we observed a more important effect in WT animals and cells in comparison with 3TG ones. This fact is certainly due to the combined effect of soluble TNF neutralization and tmTNF reverse signaling. This leads to the interpretation that tmTNF reverse signaling is not the principal or the only mechanism of TNF blockers but a complementary mechanism to soluble TNF neutralization. As tmTNF cell surface expression and signalization have been described to be related to an efficient clinical response to TNF blockers (Kadijani et al., 2017; Meusch et al., 2015), we could hypothesize that this reverse signaling is an essential complementary effect for therapeutical response.

In conclusion, our data provide evidence for the involvement of tmTNF reverse signaling in the anti-TNF-mediated modulation of arthritis *in vivo* and prompt us to consider new interpretations to the mechanisms underlying the effects of TNF inhibitors in the treatment of inflammatory diseases. Our work also paves the way to studies focused on new arginase-1-dependent therapeutic target, such as tmTNF agonists.

### Limitations of the study

Although we did not see any significant differences between WT and 3TG mice in immune cell populations in the spleen and inguinal lymph nodes and we observe an equivalent capacity of anti-TNF to interact with its ligands, we are aware that 3TG mice are just a model to study tmTNF reverse signaling and are quite different from WT mice. Indeed, WT mice express soluble TNF that participate to inflammation, and the effect of anti-TNF administration is mediated not only by tmTNF reverse signaling but also by sTNF neutralization. This lack of sTNF limits also the arthritis model that we could use. Nevertheless, we could demonstrate that tmTNF reverse signaling is a complementary mechanism of anti-inflammatory effect of anti-TNF therapies.

### Resource availability

#### Lead contact

Information and requests for resources should be directed to and will be fulfilled by the lead contact, Dr. Rauwel Benjamin (benjamin.rauwel@inserm.fr) or Dr. Degboé Yannick (yannick.degboe@inserm.fr)

#### Materials availability

This study did not generate new unique reagents.

#### Data and code availability

All relevant data are available from the lead contact upon request.

## Methods

All methods can be found in the accompanying Transparent methods supplemental file.

## Supplemental information

Document S1. Transparent methods and Figures S1–S7
