# Supplementary material for: Evidence for tmTNF reverse signaling in vivo: Implications for an arginase-1-mediated therapeutic effect of TNF inhibitors during inflammation
Source: iScience. 2021 Mar 21;24(4):102331. doi: 10.1016/j.isci.2021.102331 (PMC8050384; doi:10.1016/j.isci.2021.102331)
Supplement: Document S1. Transparent methods and Figures S1–S7 [file mmc1.pdf]

iScience, Volume *24*

Supplemental information

Evidence for tmTNF reverse signaling *in vivo*: Implications for an arginase-1-mediated therapeutic effect of TNF inhibitors during inflammation

Katy Diallo, Numa Simons, Souraya Sayegh, Michel Baron, Yannick Degboé, Jean-Frédéric Boyer, Andrey Kruglov, Sergei Nedospasov, Julien Novarino, Meryem Aloulou, Nicolas Fazilleau, Arnaud Constantin, Alain Cantagrel, Jean-Luc Davignon, and Benjamin Rauwel

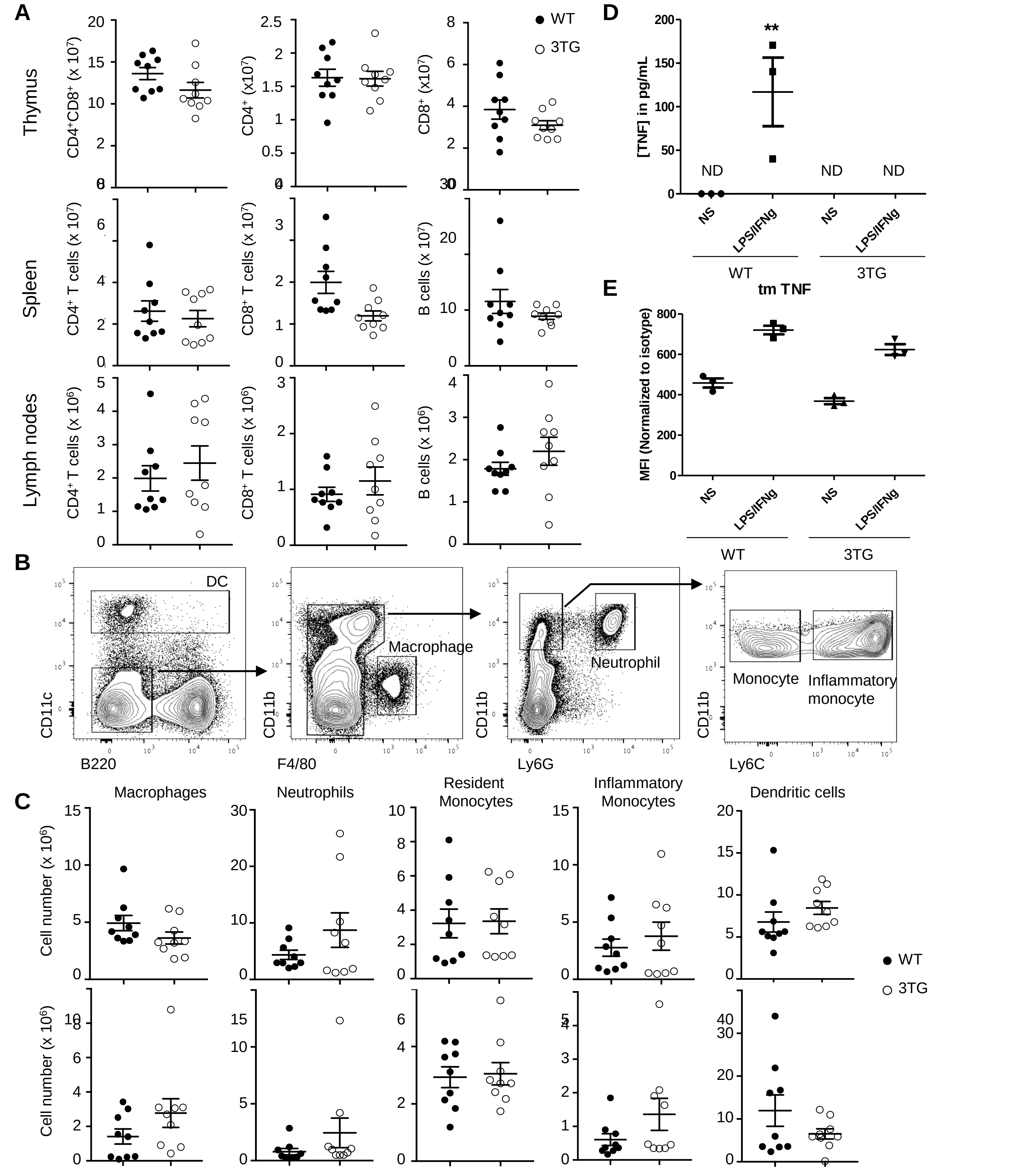

<u>Supplementary Figure 1:</u> Triple transgenic mouse tmTNF model invalidated for TNFR1/TNFR2, related to figure 1 to 6. (*A*) Cellularity of double positive $CD4^+CD8^+$, simple positive $CD4^+$ and $CD8^+$ thymocytes (Upper panel), cellularity of $CD4^+$ T cells, $CD8^+$ T cells and B cells in the spleen (middle panel) and inguinal lymph nodes (iLN) (lower panel) **(B)** Contour plots and gating strategy used for the identification of major immune cell populations in mouse spleen. Gates containing a single cell population are labeled with the included cell type **(C)** Cellularity of dendritic cells, Macrophages Neutrophils, inflammatory monocytes and resident monocytes in spleen (upper panel) and iLN (lower panel). n= 9 for each genotype. Data shown are representative from two independent experiments. Data are presented as mean ± SEM. **(D, E)** WT and 3TG BMDM were differentiated after 7 days with recombinant M-CSF (50 ng/ml). Macrophages were then polarized into pro-inflammatory M1 macrophage during 24 hours with LPS (100 ng/ml) and IFN-g (25 ng/ml). Concentration of soluble TNF in supernatant was quantified by ELISA **(D)** and tmTNF surface expression by Flow cytometry analysis **(E)**. ELISA data represent mean±SEM of cytokine concentration in pg/mL (ND=non detectable, n=3, **p<0.01, Mann-Whitney U test). Cytometry data are presented as dot-plot with mean±SEM of MFI normalized to isotype (n=3, Mann-Whitney U test).

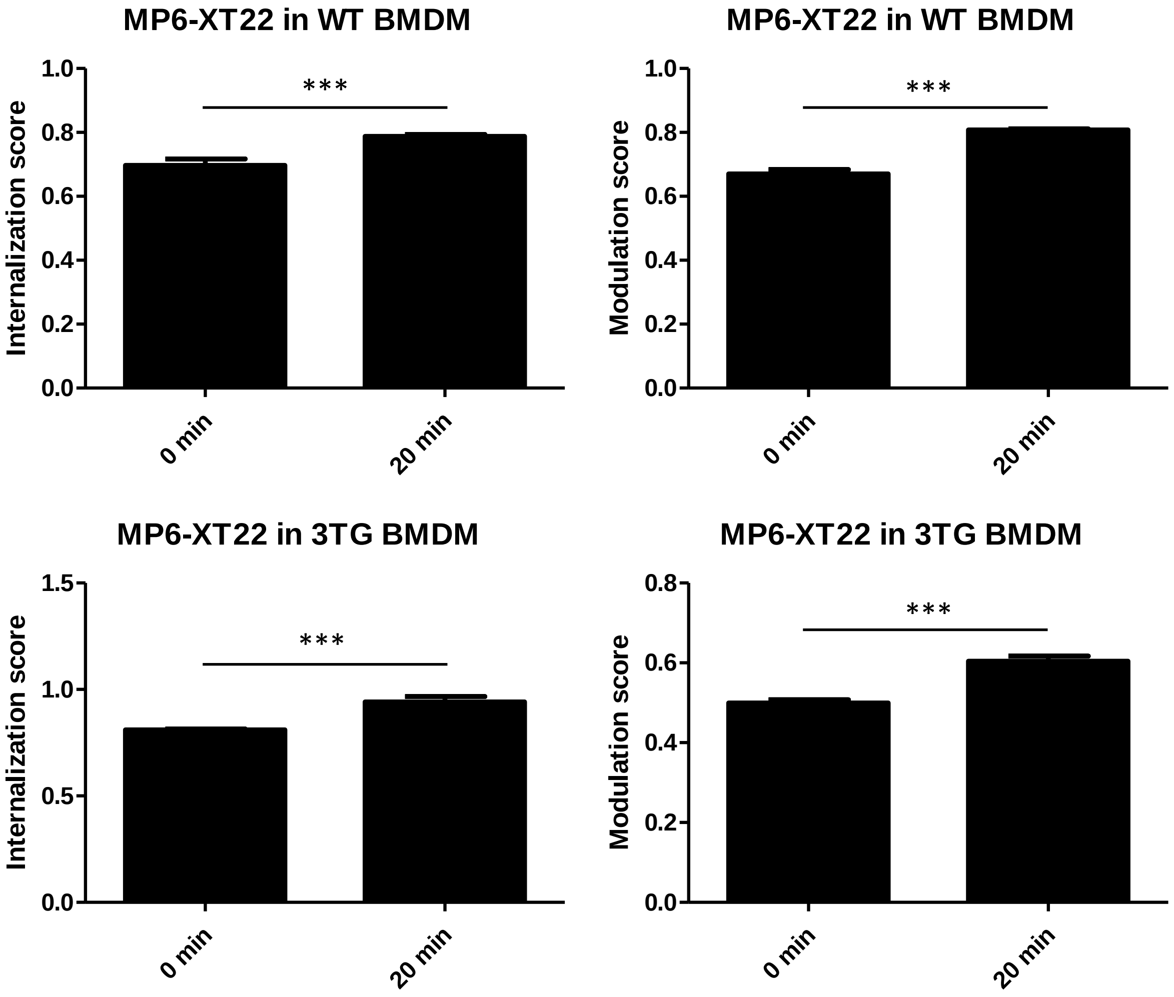

Supplementary Figure 2. Internalization of anti-TNF antibody (MP6-XT22) through its interaction with tmTNF suggests reverse signaling in macrophages, related to figure 1. Non-polarized BMDM were stimulated with LPS (50ng/mL) 30 minutes prior to staining in presence of Fc blocker with MP6-XT22-dyelight488 in WT **(A)** and 3TG **(B)** during 20 minutes at 4° (0 min) or 37°C (20min). Internalization and modulation scores were analyzed by imaging cytometry (ISX). Data are presented as mean±SEM of internalization or modulation scores (**$p<0.01$, ***$p<0.001$, n>500 events, Student's ttest). Images are representative of 3 independent experiments with more than 500 events analyzed each time.

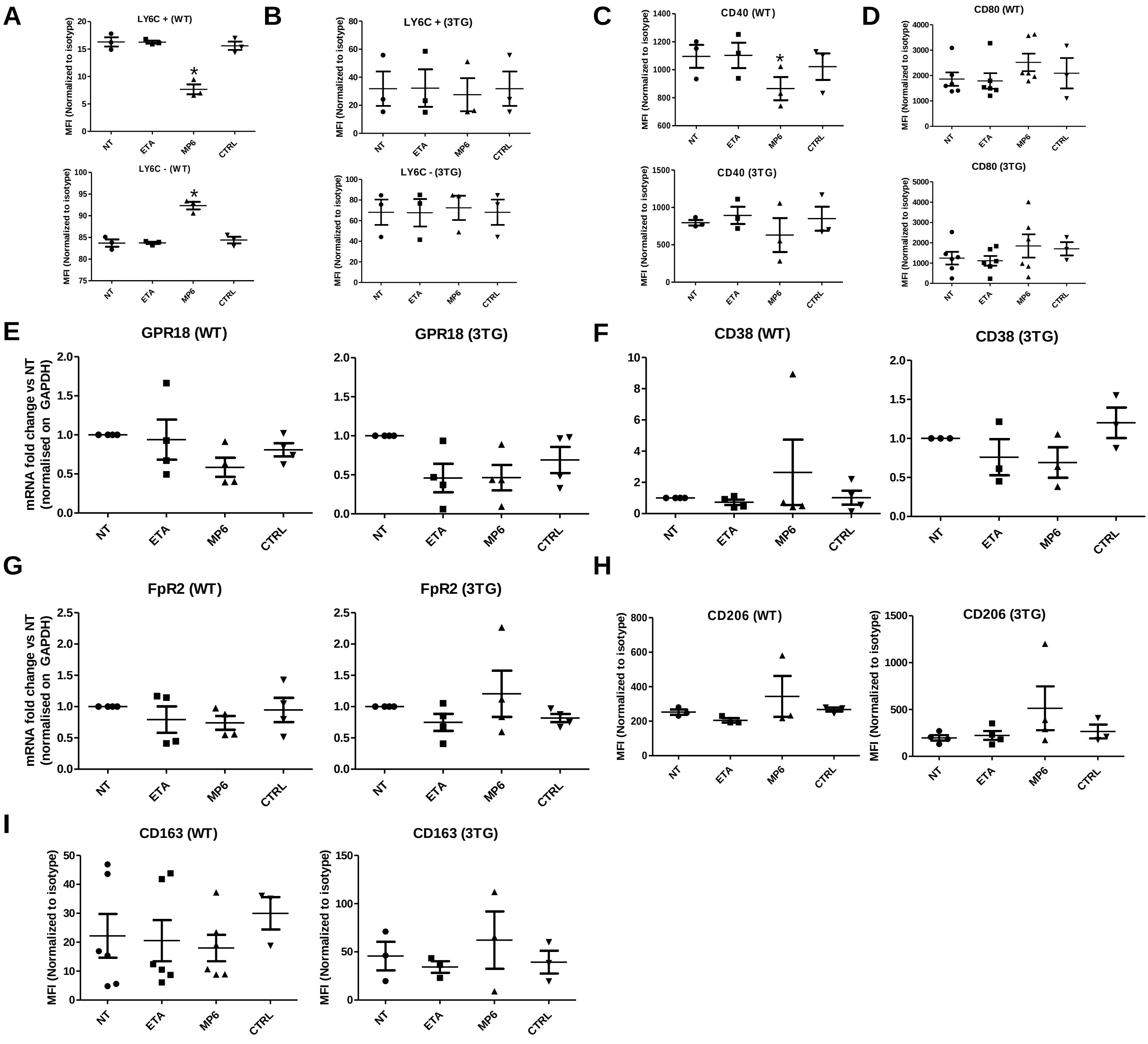

Supplementary Figure 3. Effect of tmTNF reverse signaling on macrophage polarization in vitro, related to figure 2 and 4. WT and 3TG BMDM were differentiated–after 7 days with recombinant M-CSF (50 ng/ml) in the presence or not (NT) to 10 µg/ml etanercept (ETA), anti-TNF antibody (MP6-XT22) or IgG1 control (CTRL) or left untreated (NT). Macrophages were then polarized into pro-inflammatory M1 macrophage during 24 hours with LPS (100 ng/ml) and IFN-g (25 ng/ml) in the presence or not (NT)-of fresh ETA, MP6-XT22 or CTRL. Flow cytometry analysis were performed to assess the surface expression of pro-inflammatory (Ly6C, CD40, CD80) **(A, B, C, D)** or pro-resolutive markers (CD206, CD163) **(H, I)**. Cytometry data are presented as dot-plot with mean±SEM of MFI normalized to isotype (n=3 for Ly6c, CD40, CD206, and CD163, n=6 for CD80 and CD163 in WT BMDM, *p<0.05, Mann-Whitney U test performed). RNA were extracted and pro-inflammatory (*gpr18* **E**, *cd38* **F** and *fpr2* **G**) mRNA expression was analyzed by RT-qPCR in 3TG and WT BMDM. Data are presented as mean±SEM of mRNA fold change vs NT normalized on *gapdh*. (n=4, Mann-Withney U test).

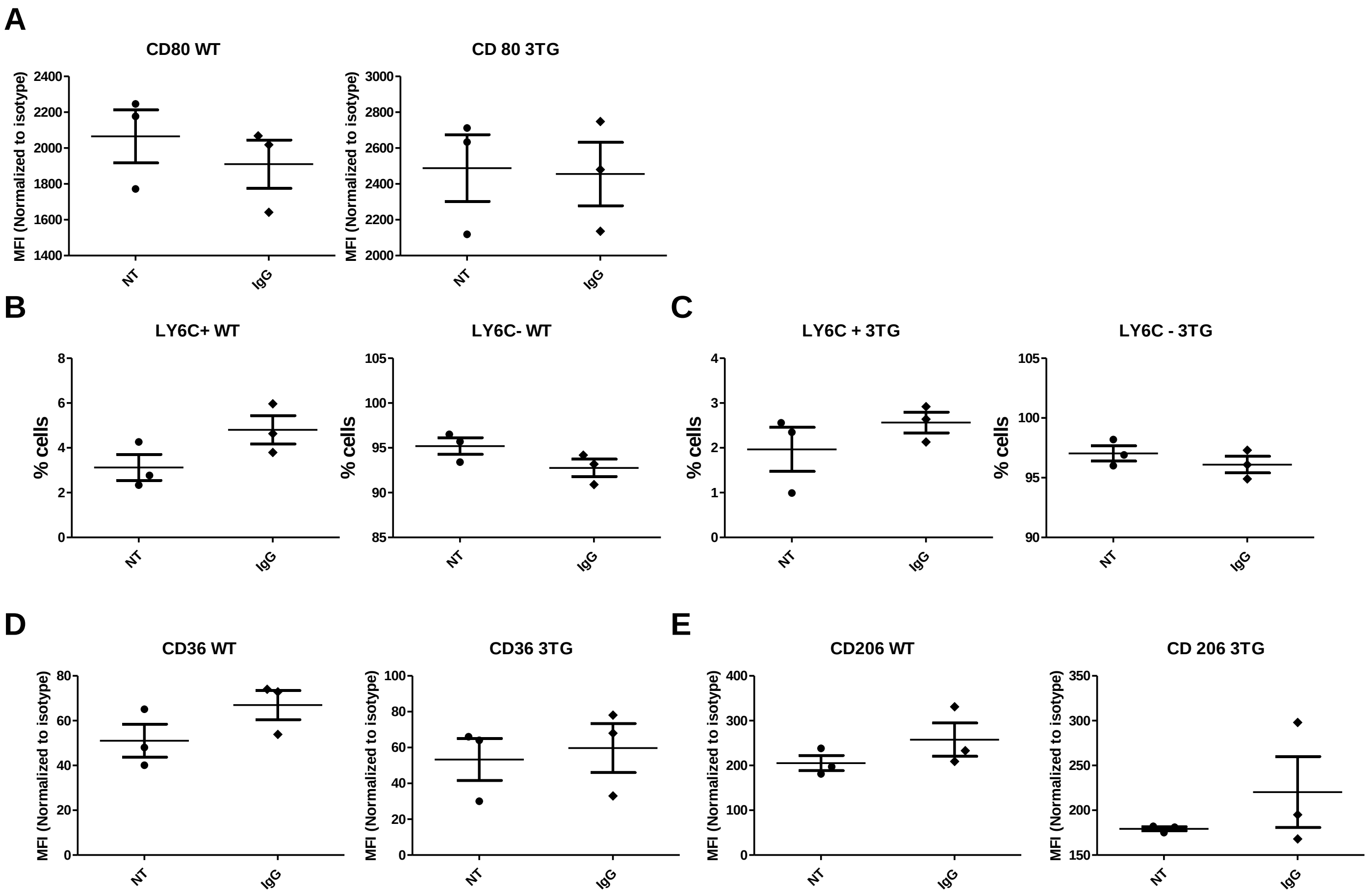

Supplementary Figure 4. Effect of tmTNF reverse signaling on macrophage polarization *in vitro*, related to figure 2 and 4. WT and 3TG BMDM were differentiated–after 7 days with recombinant M-CSF (50 ng/ml) in the presence or not (NT) to 10 µg/ml rat IgG control (IgG). Macrophages were then polarized into pro-inflammatory M1 macrophage during 24 hours with LPS (100 ng/ml) and IFN-g (25 ng/ml) in the presence or not (NT)–of fresh IgG. Flow cytometry analysis were performed to assess the surface expression of pro-inflammatory (CD80, Ly6C) **(A, B, C)** or pro-resolutive markers (CD36, CD206) **(D, E)**. Cytometry data are presented as dot-plot with mean±SEM of MFI normalized to isotype or % of cells for Ly6C (n=3 Mann-Whitney U test performed).

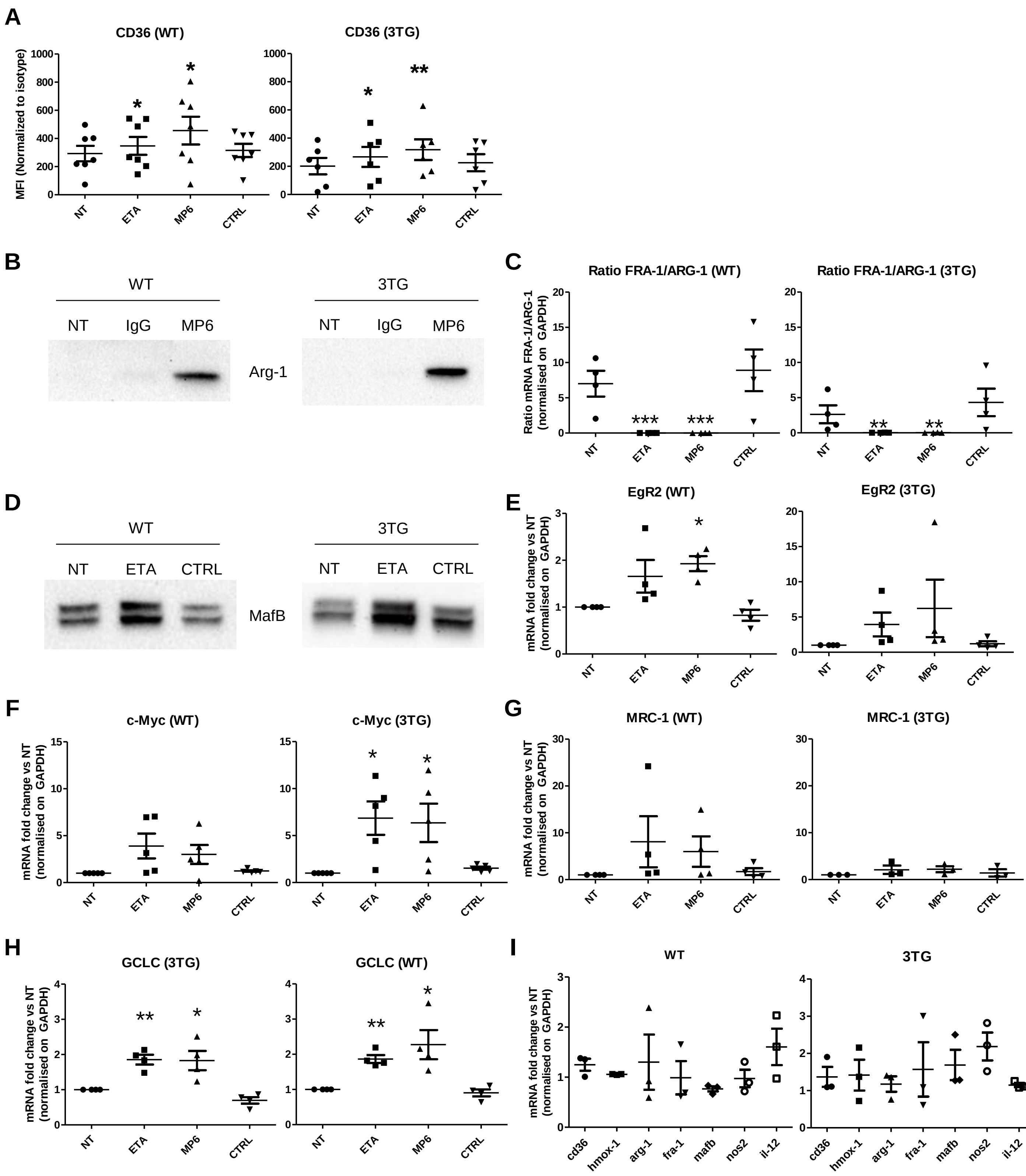

Supplementary Figure 5. Effect of tmTNF reverse signaling on macrophage polarization in vitro, related to Figure 2 and 4. WT or 3TG BMDM were obtained during 7 days of differentiation with recombinant M-CSF (50 ng/ml) in presence or absence (NT) of 10 µg/ml of etanercept (ETA), anti-TNF antibody (MP6-XT22), human control IgG1 (CTRL) or rat control IgG (**IgG, B, I)** prior to being polarized into pro-inflammatory M1 macrophage during 24 hours with LPS (100 ng/ml) and IFN-g (25 ng/ml) in presence or not (NT) with fresh ETA, MP6-XT22 or CTRL. Flow cytometry analysis were performed to assess the surface expression pro-resolutive marker CD36 **(A)**. Cytometry data are presented as dot-plot with mean±SEM of MFI normalized to isotype (n=6 in 3TG, n=7 in WT, *p<0.05, Mann-Whitney U test performed). **(B, C, D, E, F, G, H)** RNA were extracted and ratio of *fra-1/arg1* ***(C)***, *c-myc* **(E)**, *mrc-1* **(F)** *egr-2* ***(G)*** and *gclc* **(H)**, mRNA expression analyzed by RT-qPCR in 3TG and WT BMDM. Data are presented as mean±SEM of mRNA fold change vs NT normalized on gapdh. (n=4, *p<0.05, **p<0.01, Mann-Whitney U test performed). **(B, C)** Protein expression of total ARG-1 and nuclear MAFB were assessed by western-blot analysis. Results were normalized on total amount of protein with stain-free gel assay. Images are representative of 3 independent experiments.**(I)** *cd36, hmox-1, arg1, fra-1, mafb, iNos and il-12* mRNA expression analyzed Data are presented as mean±SEM of mRNA fold change vs NT normalized on gapdh. (n=3, Mann-Whitney U test performed).

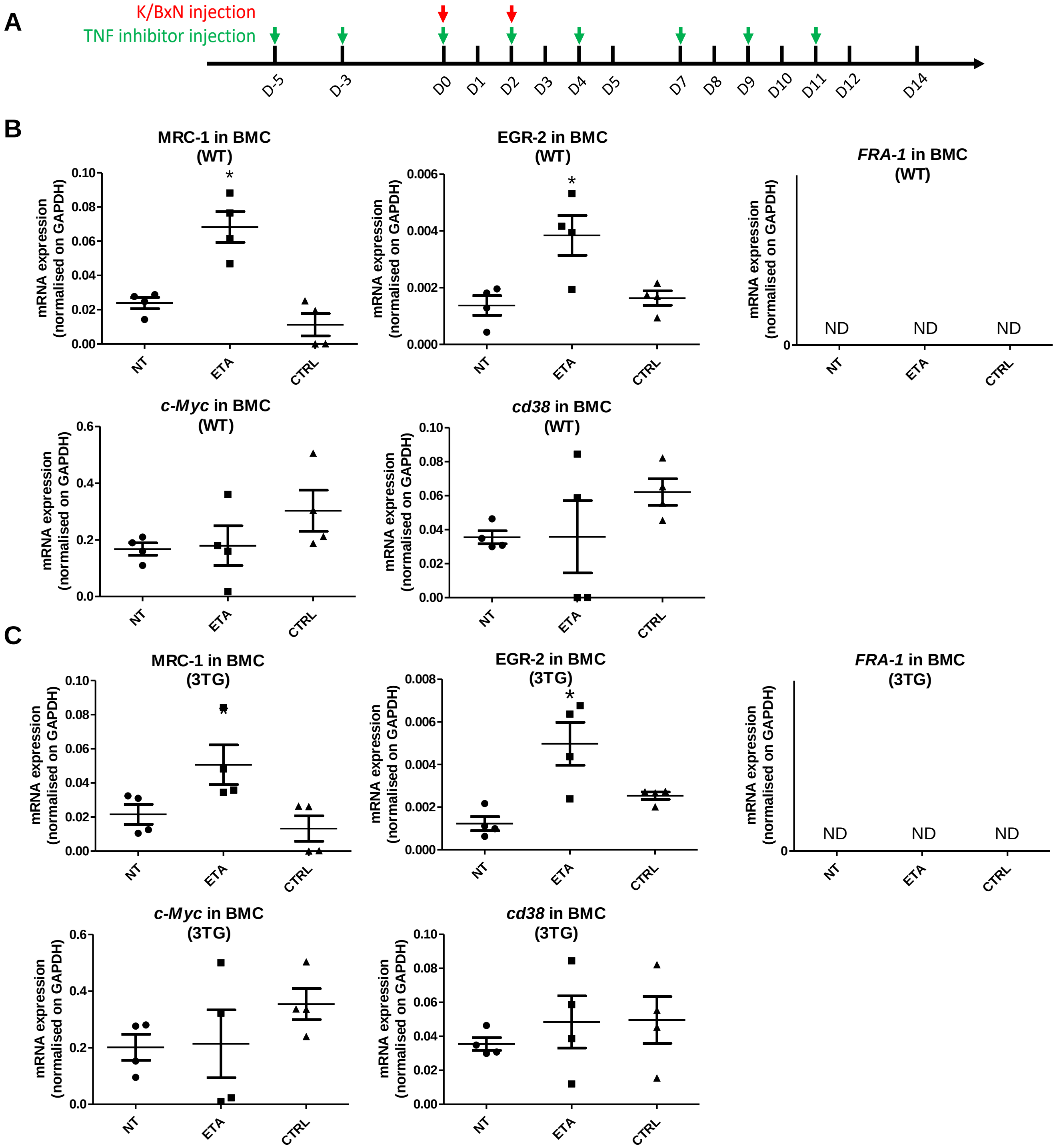

Supplementary Figure 6. In vivo arthritis experiments in WT and 3TG mice, related to Figure 5 and 6. (A) Experimental protocol of arthritis. 8-week-old 3TG or WT mice were injected at days 0 and 2 intraperitoneally with 200 µl of 60-week-old K/BxN mice serum to induce arthritis. Mice were injected 5 and 3 days prior to inducing arthritis with 10 mg/kg of anti-TNF (ETA or MP6-XT22) or control IgG1 (CTRL) and at days 0, 2, 4, 7, 9 and 11. **(B, C)** At day 0, Bone marrow from 4 mice of each group (NT, ETA and CTRL) were collected and mRNA from precursor cells extracted. Expression of *mrc-1*, *egr-2*, *fra-1*, *c-myc* and *cd38* in WT **(B)** or 3TG (**C**) mice were analyzed by RT-qPCR. Data represent mean±SEM of mRNA expression normalized on *gapdh* expression (n=4). Statistics analysis were performed with Mann-Withney U test. ND= non detectable.

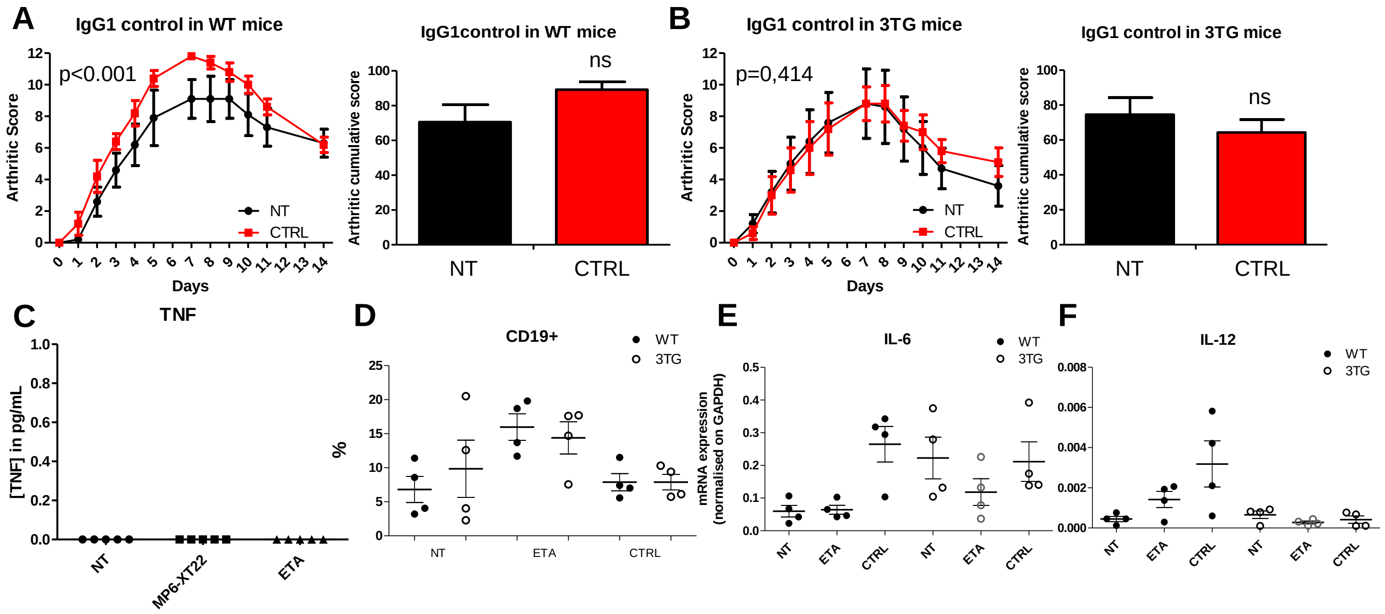

Supplementary Figure 7. In vivo arthritis experiments in WT and 3TG mice, related to Figure 5 and 6. (A, B) Clinical effect of IgG1 control antibody (CTRL, 10 mg/kg) on the development of arthritis (arthritic score) in the 3TG **(A)** or WT **(B)** K/BxN serum–transferred mice (n= 5 per group). Control (NT): untreated K/BxN serum–transferred mice. Results are presented as mean arthritic score during 14 days after K/BxN injection (left panel) and arthritic cumulative score over these 14 days (right panel). Data represent mean ± SEM. P value for arthritis score was calculated by repeated measurements two-way ANOVA test. P value for Arthritic cumulative score was calculated with Student's t-test (ns, p>0.05). **(C)** Concentrations of TNF in blood sample of 3TG arthritic mouse were quantified by Cytometric Bead Array 7 days after K/BxN first injection. Data represent mean±SEM of cytokine concentration (n=5, Student's t-test). **(D, E, F)** Mice were sacrificed at day 7 and joints were dissected. **(D)** Flow cytometry analysis of % of B lymphocytes. RT-qPCR analysis of *il-6* **(E)** and *IL-12p40* ***(F)*** mRNA expression in WT and 3TG joints. Data represent mean±SEM % of leaving cells or mRNA expression normalized on GAPDH (n=4, *p< 0.05 as calculated with Mann-Whitney U test).

Transparent Methods

Mice

Triple transgenic mice (3TG: TNFR1$^{-/-}$, TNFR2$^{-/-}$, tmTNF$^{KI/KI}$) in C57BL/6 genetic background were specifically obtained by crossing existing TNFR1 and TNFR2 KO mice from Jackson laboratories to obtain TNFR1/R2 double KO that we crossed with tmTNF KI mice from our collaborators laboratory(Ruuls et al., 2001). 3TG mice were housed in a specific pathogen-free environment and cared for in accordance with European institutional guidelines (http://eur-lex.europa.eu). *In vivo* experiments were performed under the protocol CEEA-122 2014-62, authorized by the "Comité d'éthique en matière d'expérimentation animale CEEA122-US006/CREFRE".

Organ and cell isolation

Single-cell suspensions were prepared by standard mechanical disruption for thymus and filtered through a nylon mesh. Spleen and inguinal lymph node (iLN) were first enzymatically digested Liberase (50ug/ml, Roche) and DNase I (10ug/ml, Roche Molecular Biochemicals) for 20 min at 37℃. Cells were counted and surface stained with: anti-B220-PECy7 (RA3-6B2, BioLegend), anti-Ly6C-BV711 (HK1.4, Biolegend), anti-CD8-PE-CF594 (53-6.7, BD Biosciences), Anti-CD8-PE-CF594 (53-6.7, BD Biosciences), Ly6G-FITC (1A8, BD Biosciences), anti-CD11c-PE-CF594 (HL3, BD Biosciences), Streptavidin-BV605 (BD Biosciences), anti-CD3-V500 (500A2, BD Biosciences), anti-CD4-PerCP-Cyanine5.5 (RM4-5, eBioscience), anti-CD11b-PE-Cy5 (M1/70, eBioscience) and F4/80-biot (BM8, eBioscience). Cells were stained with Fixable Viability Dye eFluor506 (eBioscience, Cat#65-0866) or Dye eFluor450 (eBioscience, Cat#65-0863). Labelled cells were acquired and analyzed using an LSRII flow cytometer (BD Biosciences, San Jose, CA) and FlowJo software (Tree Star, Ashland, OR). Doublets and dead cells were excluded using appropriate FSC/SSC gates.

Bone marrow derived macrophages (BMDM)

To generate BMDM, bone marrow cells from femurs and tibias of mice were harvested using aseptic techniques. Marrow cores were flushed into sterile tubes using syringes fitted with 23 gauge needles and filled with PBS. Cells were filtrated on 100 µm nylon cell strainer and red blood cells were lysed in lysis

buffer (0.15 m NH4Cl, 10 mm KHCO3, and 0.1 mm Na2EDTA, pH 7.4). Cells were washed once in PBS then plated and cultured at a density of 5-6x106cells/well (6-well plate, Falcon poly-styrene) in Dulbecco's Modified Eagle Media (DMEM, Invitrogen) supplemented with 10% heat-inactivated fetal bovine serum (FBS) (Life Technologies), 1% penicillin/streptomycin (Invitrogen) and recombinant M-CSF (50 ng/mL, peprotech), in the presence or absence of 5 µg/mL of soluble TNF-R2 (Etanercept, ETA), anti-murine TNF rat antibody (MP6-XT22) or anti-human IL-17 monoclonal antibody (Secukinumab, CTRL) as a non-relevant antibody control. After 7 days of differentiation, fresh ETA, MP6-XT22 or CTRL were added to media and cells were classically polarized into macrophages M1 in the presence of LPS (100 ng/ml, Sigma-Aldrich L2880) + IFN-γ (25 ng/mL, peprotech) for 24 hours.

Arthritis model

8-weeks-old 3TG or WT male mice were injected at days 0 and 2 intraperitoneally with 200 µl of 60-week-old K/BxN mice serum to induce arthritis. Soluble TNF receptor 2 (Etanercept, ETA, Pfizer), anti-mouse TNF rat antibody (MP6-XT22) or IgG1 control antibody (CTRL, Secukinumab, Novartis Pharma) were injected intraperitoneally at 10 mg/kg, 5 and 3 days prior to the first K/BxN serum injection and at days 0, 2, 4, 7, 9 and 11. Mice were sacrificed at day 0 or 14 days after the first K/BxN injection. Each joint was examined daily for swelling and redness. Severity of arthritis in K/BxN-injected mice was assessed macroscopically in a blinded fashion for each paw per mouse with a three-grade score (Grade 0 = normal; grade 0.5 = swelling of fingers; grade 1 = light swelling of the joint and/or redness of the footpad; grade 2 = obvious swelling of the joint and grade 3 = severe swelling of the joint with redness of the footpad). A severity score was calculated for the four limbs (maximum 12 points for individual mice). Cumulative arthritis score for all mice was calculated at day 14. Swelling of the two hindpaws was measured with a digital caliper and averaged. Blood samples were collected at the peak of arthritis score, 7 days after K/BxN serum injection. Progenitor cells from bone marrow were collected at day 0 to study the effect of anti-TNF pre-injection. At day 7, mice were sacrificed and knees and ankles joints were dissected mechanically in presence of DNase and Collagenase D. Purified cells were then separated in two, one half for flow cytometry analysis and one half for RT-qPCR analysis.

Internalization assay

We used imaging cytometry to determine if soluble TNF receptor 2 (Etanercept, ETA, Pfizer) molecules bind tmTNF and are internalized into BMDM. BMDM were stimulated for 30 min with LPS (50 ng/mL) to increase tmTNF cell surface expression. Cells were then harvested, resuspended in PBS containing 5% FCS and incubated in the presence of anti-H-2 (I-A / I-E, 15-532-81, ebioscience) or ETA conjugated to PE-Cy5 or DyeLight488 respectively. H-2 was used as a non-internalized control. Staining was operated 30 min at 37°C to permit internalization. A control staining at 4°C to block internalization was performed in parallel. Cells were washed and resuspended in PBS, 5mM EDTA prior to analysis by Image Stream X Mark II (Merck Millipore). Internalization and modulation scores of tmTNF/soluble receptors were calculated through the ratio of intracellular fluorescence to Total fluorescence. Modulation score indicates the concentration of fluorescence in spots as opposed to scattered signal.

RNA extraction and RT-qPCR

BMDM were harvested and analyzed after 7 days of differentiation and 24 hours of M1 polarization. RNA was extracted by using High Pure RNA Isolation Kit (Roche, and reverse transcribed with RevertAid Reverse Transcriptase (ThermoFisher Scientific, Waltham, MA, USA) according to the manufacturer's instructions. All qPCRs were performed with SYBR green mix (Roche, Switzerland).

All RT-qPCR were performed with the following primers:

gapdh	Forward	5'-TTCACCACCATGGAGAAGG-3'
	Reverse	5'-CACACCCATCACAAACATGG-3'
ho-1	Forward	5'-GGTGATGGCTTCCTTGTACC-3'
	Reverse	5'-AGTGAGGCCCATACCAGAAG-3'
cd36	Forward	5'-TCCTCTGACATTTGCAGGTCTATC-3'
	Reverse	5'-AAAGGCATTGGCTGGAAGAA-3'

gclc	Forward	5'-GCACGGCATCCTCCAGTTCCT-3'
	Reverse	5'-TCGGATGGTTGGGGTTTGTCC-3'
cd38	Forward	5'-TCAGCCACTAATGAAGTTGGGA-3'
	Reverse	5'-CTGGACCTGTGTGAACTGATGG-3'
gpr18	Forward	5'-GACAGACAGGAGGTTCGACATACA-3'
	Reverse	5'-ACCGAGGTGTGGGTCTCCTTATGT-3'
fpr2	Forward	5'-CTGAATGGATCAGAAGTGGTGG-3'
	Reverse	5'-CCCAAATCACTAGTCCATTGCC-3'
egr-2	Forward	5'-GCCAAGGCCGTAGACAAAATC-3'
	Reverse	5'-CCACTCCGTTCATCTGGTCA-3'
c-myc	Forward	5'-CGGACACACAACGTCTTGGAA-3'
	Reverse	5'-AGGATGTAGGCGGTGGCTTTT-3'
il-6	Forward	5'-TACCCCAATTTCCAATGCTC-3'
	Reverse	5'-TCTTGGTCCTTAGCCACTCC-3'
arg1	Forward	5'-GAATCTGCATGGGCAACC-3'
	Reverse	5'-GAATCCTGGTACATCTGGGAAC-3'
il-10	Forward	5'-CAGAGCCACATGCTCCTAGA-3'
	Reverse	5'-TGTCCAGCTGGTCCTTTGTT-3'
il-12	Forward	5'-TTGCTGGTGTCTCCACTCAT-3'
	Reverse	5'-GGGAGTCCAGTCCACCTCTA-3'
mrc1	Forward	5'-CCACAGCATTGAGGAGTTTG-3'

	Reverse	5'-ACAGCTCATCATTTGGCTCA-3'
nos2	Forward	5'-CCGGAGCCTTTAGACCTCA-3'
	Reverse	5'-TTCAGCCTCATGGTAAACACA-3'
fra-1	Forward	5'-CCCAGTACAGTCCCCCTCA-3'
	Reverse	5'-TCCTCCTCTGGGCTGATCT-3'
il-1β	Forward	5'-TGAAAGACGGCACACCCA-3'
	Reverse	5'-AAACCGCTTTTCCATCTTCTTCT-3'

Western Blot Analysis

Cell lysates were subjected to SDS/PAGE on 4–15% Mini-Protean TGX Stain-Free Gels (BioRad, Hercules, CA, USA). After transferring on 0.22 µm nitrocellulose membrane, proteins were revealed using specific antibodies (MafB rabbit monoclonal antibody, clone BLR046F, Bethyl laboratories, Montgomery, TX, USA) and anti-rabbit HRP-linked polyclonal antibodies (Cell Signaling, Danvers, MA, USA). Protein expression was normalized on total protein amount using stain-free technology following manufacturer's instructions.

Flow cytometry

Evaluation of the effect of reverse signaling on M1 polarization of BMDM was assessed by studying the expression of surface markers by flow cytometry. The cells were stimulated for 24 hours with 100 ng/ml lipopolysaccharide (LPS) and 25 ng/ml interferon γ (IFN γ). For extracellular labeling, non-specific Ab binding was blocked with addition of blocking buffer PBS 20% SVF. Cells were washed prior to being labeled with antibodies described in supplemental materials. F4 / 80 FITC (clone BM8, Biolegend), CD36 PE (ME542 clone, Santa Cruz Biotechnolgy), CD206 (clone C068C2, Biolegend), CD163 PE (clone TNKUPJ, Invitrogen), CD80 FITC (clone 16-10A1, Biolegend), Ly6C PE (clone AL21, BD Biosciences). Flow cytometry was performed by using a MACSQuant analyzer 10 (Miltenyi) and data were analyzed using FlowJo software.

Cytokine concentration analysis

IL-6, IL-10, TNF and IL12p70 concentrations in blood samples and 24h M1 polarized BMDM supernatants were analyzed by Cytometric Bead Array (CBA, BD Biosciences) according to manufacturer's instructions. Data was acquired on an LSRII cytometer (BD Biosciences) and analyzed using FCAP Array v3 software (BD Biosciences). 24 hours secretion kinetic of IL-1β, TNF and IL-10 in M1 polarized BMDM were analyzed by ELISA (Biolegend).

Statistical analysis

All data were analyzed with GraphPad Prism5. Normality was tested by Agostino and Pearson test. *In vitro* data were analyzed with Student's T-test or Mann-Whitney U-test. *In vivo* arthritis experiments data were analyzed with repeated-measurements two-way ANOVA test. Data are represented as mean ± SEM, and $p<0.05$ (two-tailed) was considered to be statistically significant.
